# Activation of Nrf2 by Lithospermic Acid Ameliorates Myocardial Ischemia and Reperfusion Injury by Promoting Phosphorylation of AMP-Activated Protein Kinase *α* (AMPK*α*)

**DOI:** 10.3389/fphar.2021.794982

**Published:** 2021-11-26

**Authors:** Min Zhang, Li Wei, Saiyang Xie, Yun Xing, Wenke Shi, Xiaofeng Zeng, Si Chen, Shasha Wang, Wei Deng, Qizhu Tang

**Affiliations:** ^1^ Department of Cardiology, Renmin Hospital of Wuhan University, Wuhan, China; ^2^ Hubei Key Laboratory of Metabolic and Chronic Diseases, Wuhan, China; ^3^ Department of Pediatrics, Renmin Hospital of Wuhan University, Wuhan, China

**Keywords:** lithospermic acid, myocardial ischemia-reperfusion injury (MIRI), Nrf2, oxidative stress, ampk*α*

## Abstract

**Background:** As a plant-derived polycyclic phenolic carboxylic acid isolated from *Salvia miltiorrhiza*, lithospermic acid (LA) has been identified as the pharmacological management for neuroprotection and hepatoprotection. However, the role and mechanism of lithospermic acid in the pathological process of myocardial ischemia-reperfusion injury are not fully revealed.

**Methods:** C57BL/6 mice were subjected to myocardial ischemia and reperfusion (MI/R) surgery and pretreated by LA (50 mg/kg, oral gavage) for six consecutive days before operation. The *in vitro* model of hypoxia reoxygenation (HR) was induced by hypoxia for 24 h and reoxygenation for 6 h in H9C2 cells, which were subsequently administrated with lithospermic acid (100 μM). Nrf2 siRNA and dorsomorphin (DM), an inhibitor of AMPKα, were used to explore the function of AMPK*α*/Nrf2 in LA-mediated effects.

**Results:** LA pretreatment attenuates infarct area and decreases levels of TnT and CK-MB in plasm following MI/R surgery in mice. Echocardiography and hemodynamics indicate that LA suppresses MI/R-induced cardiac dysfunction. Moreover, LA ameliorates oxidative stress and cardiomyocytes apoptosis following MI/R operation or HR *in vivo and in vitro*. In terms of mechanism, LA selectively activates eNOS, simultaneously increases nuclear translocation and phosphorylation of Nrf2 and promotes Nrf2/HO-1 pathway *in vivo and in vitro*, while cardioprotection of LA is abolished by pharmacological inhibitor of AMPK or Nrf2 siRNA in H9C2 cells.

**Conclusion:** LA protects against MI/R-induced cardiac injury by promoting eNOS and Nrf2/HO-1 signaling via phosphorylation of AMPK*α*.

## 1 Introduction

Myocardial ischemia-reperfusion (MI/R) injury has a significant impact on the prognosis of patients with revascularization (Jennings, 2013). Moreover, MI/R injury largely contributed to cardiac dysfunction through additional oxidative stress and apoptosis in the heart (Lu et al., 2018; Chen et al., 2019). Therefore, reducing MI/R injury following acute myocardial infarction (AMI) greatly improves morbidity and mortality. Unfortunately, the pathogenesis and molecular mechanisms of MI/R injury in the heart are poorly understood (Lejay et al., 2016). A considerable number of patients with MI/R injury still have a poor prognosis after conventional treatment. Hence, a better understanding of the mechanism of MI/R injury would make it possible to propose more effective interventions.

As a well-known Chinese herbal medicine, *Salvia miltiorrhiza* has been widely utilized to prevent cardiovascular diseases (CVDs) in China and Asia (Wang et al., 2017; Jia et al., 2019). Lithospermic acid (LA), a catechol derivative extracted from *Salvia miltiorrhiza*, is a natural compound with diversified biological activities. Cheng et al. revealed that LA attenuated diabetes and target organ damage in rats (Jin et al., 2014). Moreover, LA has been suggested to prevent Parkinson’s disease by suppressing apoptosis and inflammation in the nervous system (Lin et al., 2015). In addition, LA has been shown to exert a hepatoprotective effect against carbon tetrachloride (CCl_4_)-induced hepatic oxidative damage (Chan and Ho, 2015). Importantly, previous clinical trials showed that LA injection improved coronary heart diseases angina pectoris (Zhang et al., 2006). However, the exact contribution of LA in MI/R injury following AMI remains largely elusive.

As a master transcription factor expressed in multiple tissues, nuclear factor erythroid 2-related factor 2 (Nrf2) is implicated in antioxidant defense mechanisms in the myocardium, which is usually activated by increased reactive oxygen species (ROS) production (Chen and Maltagliati, 2018; Vashi and Patel, 2020). Nrf2 upregulates detoxicant genes in response to the stimulatory signal, leading to cardioprotection (Boo, 2020). Accumulating evidence reveals that Nrf2 reduces oxidative stress and myocardial inflammation in heart tissues by activation of the Nrf2-antioxidant response element (ARE) pathway (Buendia et al., 2016; Guo and Mo, 2020). Previous studies have also uncovered the cardioprotection of the Nrf2 pathway. Most recently, Guo et al. reported that inhibition of the Nrf2-ARE pathway promoted oxidative stress-induced necrosis and ischemia/reperfusion injury (Guo et al., 2020). Furthermore, activation of the Nrf2-ARE pathway suppresses oxidative stress, and ameliorates isoproterenol-mediated pathological cardiac hypertrophy progression (Velusamy et al., 2020). Additionally, phosphorylation of Nrf2 has been suggested to inhibit oxidative stress and apoptosis in human dermal fibroblasts (Huang et al., 2019). Manuel et al. suggested that AMP-activated protein kinase (AMPK) triggered phosphorylation of Nrf2 and promoted transactivation of antioxidative genes (Matzinger et al., 2020).

In the present study, we demonstrate that LA improves cardiac function and attenuates myocardial injury during MI/R. Moreover, LA suppresses oxidative stress and apoptosis by promoting activation of endothelial nitric oxide synthase (eNOS) and Nrf2/HO-1 pathway via phosphorylation of AMPKα in MI/R injury.Table

## 2 Methods

All animal experimental procedures followed the National Institutes of Health (NIH) guidelines and were approved by the Animal Care and Use Committee of Renmin Hospital of Wuhan University. Lithospermic acid (>98% purity, CAS. 28831-65-4) was obtained from Shanghai Winberb Medical Technology Co., Ltd (Shanghai, China).

### 2.1 Animals

C57BL/6 male mice (2 months old, 24.5 ± 2.0 g), obtained from the Institute of Laboratory Animal Science, Chinese Academy of Medical Sciences (Beijing, China), were randomly separated into 4 groups: Sham-NS (normal saline) (*n* = 10), Sham-LA (*n* = 10), MI/R-NS (*n* = 15) and MI/R-LA (*n* = 15). Mice were pretreated with LA (50 mg/kg, oral gavage) for six consecutive days before MI/R surgery or sham, and poured into the normal saline control group processing.

A myocardial ischemia-reperfusion mouse model was constructed as previously described (Fan et al., 2017). Briefly, mice were anesthetized with pentobarbital (50 mg/kg, i.p.). Afterward, mice subjected to skin preparation were intubated and connected to a small animal ventilator. Subsequently, surgical scissors were used to cut the fourth intercostal space on the left side to fully expose the heart. Then, the left anterior descending coronary artery (LAD) was ligated with 6-0 silk thread at 2 mm below the left atrial appendage. Mice were exposed to 45 min of LAD occlusion, followed by 24 h of reperfusion. Meanwhile, the small animal electrocardiogram (ECG) monitoring system was utilized to record ST-segment elevation. Finally, the rodents were euthanized with an overdose of pentobarbital (200 mg/kg, i.p.), and their hearts were harvested for further analysis.

### 2.2 Echocardiography and Hemodynamics

Mice subjected to 24 h of reperfusion were anesthetized by inhalation of 1.5–2% isoflurane (Fan et al., 2017). The cardiac structure and function were monitored using a MyLab 30CV system (Biosound Esaote, Inc.) equipped with a 15 MHz probe in a small animal ultrasound instrument. Parameters were obtained from more than three beats and then averaged. Left ventricular internal diameter at end-diastole (LVIDd), left ventricular internal diameter at end-systole (LVIDs) and left ventricular fractional shortening (LVFS) were tested. Hemodynamic parameters were obtained using a 1.4-French catheter-tip micromanometer catheter (SPR-839; Millar Instruments, Houston, TX, United States ), which was inserted into the left ventricle (LV) through the right carotid artery. Subsequently, pressure-volume parameters were recorded using an ARIA pressure-volume conductance system (MPVS-300 Signal Conditioner, Millar Instruments, Houston, TX, United States) coupled to a Power Lab/4SPA/D converter, and then analyzed by Lab Chart 8 software.

### 2.3 Histological Analysis

Double staining with Evans blue and 2,3,5-triphenyltetrazolium chloride (TTC) was utilized to determine the area at risk and infarcted area following MI/R operation. More details are provided in the supplement material. The isolated heart tissue was immobilized with 4% paraformaldehyde and dehydrated with gradient alcohol. Then, 5 μm-thick sections were paraffin-embedded. Immunohistochemistry (IHC) of 4-hydroxynonenal and Nrf2 was performed to detect oxidative stress and the location of Nrf2 in the heart. Dihydroethidium (DHE) fluorescence was also performed to assess oxidative. Moreover, a terminal-deoxynucleotidyl transferase-mediated nick end labeling (TUNEL) assay was used to determine apoptotic cells in the heart after MI/R surgery. The density of IHC and DHE staining was assessed using Image-Pro Plus version 6.0. More details are provided in the supplement material.

### 2.4 Western Blot

The heart sample was collected after reperfusion for 24 h. Protein was extracted from heart homogenates by radioimmunoprecipitation assay (RIPA) lysis buffer. Nuclear protein was obtained *in vivo* and *in vitro* using the Nuclear and Cytoplasmic Protein Extraction kit (P0028, Beyotime, Shanghai, China) and protein concentration was determined by bicinchoninic acid assay (Thermo Scientific, 23,227). Subsequently, 10% sodium dodecyl sulfate-polyacrylamide gel electrophoresis (SDS-PAGE) was performed to separate protein samples (40 μg), and then proteins were transferred to polyvinylidene difluoride (PVDF) membranes. After incubation with blocking buffer and targeted antibodies overnight, the PVDF membranes were subsequently incubated with a corresponding secondary antibody. Afterward, targeted protein bands were examined using a chemiluminescence method, and the density of target bands was evaluated by AlphaEaseFC software processing system (Bio-Rad, ChemiDoc XRS) and Image Lab software. Primary antibodies used in this study are provided in Supplement Table S1.

### 2.5 Real-Time Polymerase Chain Reaction (RT-PCR)

Total RNA was extracted from mouse hearts and H9C2 cells using Trizol reagent (Invitrogen, 15596-026) and cDNA was produced using the Transcriptor First Strand cDNA synthesis kit (04897030001, Roche Diagnostics, Basel, Switzerland). Meanwhile, SYBR Green (04707516001) was utilized to amplify transcripts, and GAPDH was the endogenous reference. All primers used are listed in Supplement Table S2.

### 2.6 Culture and Treatment of Cardiomyocytes

H9C2 cells were obtained from the Cell Bank of the Chinese Academy of Sciences (Shanghai, China) and cultured in Dulbecco’s modified Eagle’s medium (DMEM, GIBCO, C11995). Afterward, H9C2 cells in good growth condition were divided into 4 groups: Normoxia (Nor)-PBS group, Nor-LA group, hypoxia-reoxygenation (HR)-PBS group and HR-LA group. For Nor, cells were incubated under 5% CO_2_ at 37°C, whereas for HR, cells were subjected to hypoxia for 24 h and reoxygenation for 6 h with 5% CO_2_, 94% N_2_ and 1% O_2_ at 37°C in tri-gas incubators. H9C2 cells in the LA group were subjected to LA (100 μM) treatment for 24 h after Nor or HR.

For small interfering RNA (siRNA)-mediated knockdown experiments, Nrf2-siRNA (sc-156128) or Scr-siRNA was transfected with Lipofectamine 6,000 (lipo6000, Beyotime, C0526) at 40 nM concentration in culture medium following the manufacturer’s protocol. Western blot analysis was performed to assess the efficiency of knockdown. For the AMPK inhibitor, dorsomorphin (DM, 10 μM, Cat No. HY-13418A, purchased from MedChemExpression) was incubated with H9C2 cells for 18 h after Nor or HR. Immunoblotting was performed to assess the efficiency of blockage.

### 2.7 Cellular Immunofluorescence

After washing with phosphate-buffered saline (PBS), H9C2 cells were fixed by 4% paraformaldehyde for over 15 min. Afterward, permeabilization was performed with 0.1% Triton X-100 (Amresco) in PBS. Then cells were incubated with 10% goat serum and subsequently stained with anti-Nrf2 overnight at 4°C. Next, cells scrubbed with PBS were then subjected to the secondary antibody goat anti-rabbit Alexa Fluor™ 488 (Invitrogen, A10266) for 1 h. SlowFade® Gold anti-fade reagent with DAPI (Invitrogen, S36939) was utilized for mounting as previously described (Wu et al., 2018).

### 2.8 Data and Statistical Analysis

Data are expressed as mean ± standard error of the mean (SEM) and evaluated using SPSS version 22.0 (SPSS Inc, Chicago). Comparison of multiple groups was performed using one-way analysis of variance (ANOVA) and two group comparisons were analyzed by unpaired Student’s t-test. A *p*-value <0.05 was considered statistically significant.

## 3 Results

### 3.1 LA Ameliorates MI/R-Induced Myocardial Injury *in vivo*

**FIGURE 1:**
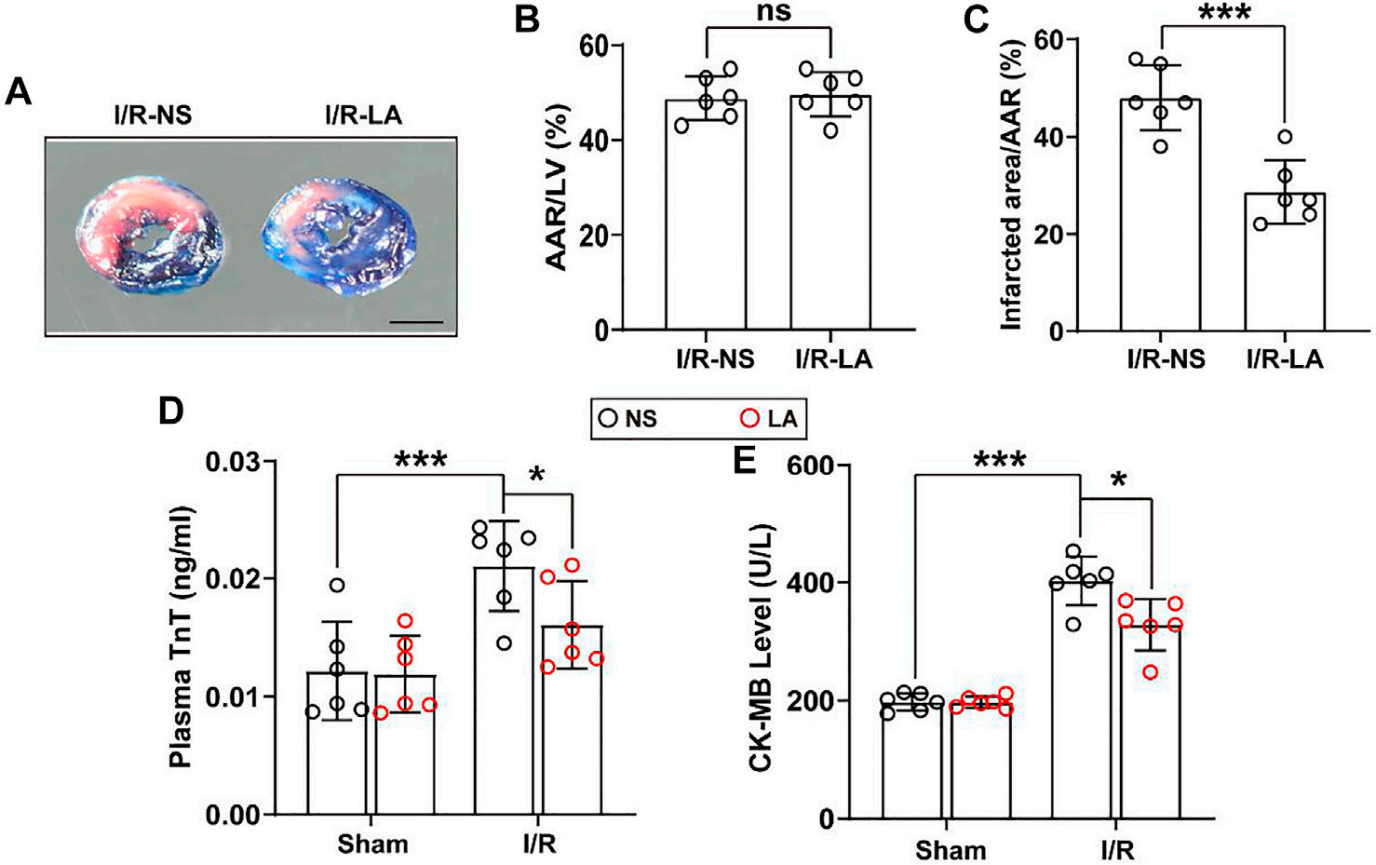
LA ameliorates MI/R-induced myocardial injury **(A)**. Left ventricular (LV) tissue sections of both LA and NS pretreated mice stained with Evans blue and 2,3,5-triphenyltetrazolium chloride (TTC) at 24 h after MI/R, in order to delineate the area at risk (AAR) and the infarcted region (Scale bar, 1 mm) **(B,C)**. The ratios of AAR/LV and infarct area/AAR were compared between LA and NS pretreated mice (*n* = 6) **(D,E)**. The enzyme activity of CK-MB and TnT in serum were accessed in LA and NS pretreated mice 24 h after MI/R operation by Elisa assay (*n* = 6). Data are presented as the mean ± SEM, with each point representing a mouse. * indicates *p* < 0.05, *** indicates *p* < 0.001, ns indicates no significance.

MI/R injury is characterized by a significant increase in oxidative stress, inflammation and apoptosis in the heart tissues (Sun et al., 2021). LA has been previously verified as a potential antioxidant drug (Kang et al., 2021). To determine whether LA might reduce myocardial injury, plasma levels of troponin T (TnT) and creatine kinase MB (CK-MB) and myocardial infarct area were measured. It was found that the myocardial infarct area was reduced following MI/R surgery, suggesting a cardioprotective effect of LA (Figures 1A–C). In addition, plasma levels of TnT and CK-MB were elevated following MI/R operation, which were significantly reduced by LA pretreatment (Figures 1D,E).

### 3.2 LA Improves MI/R-Mediated Cardiac Dysfunction *in vivo*

**FIGURE 2:**
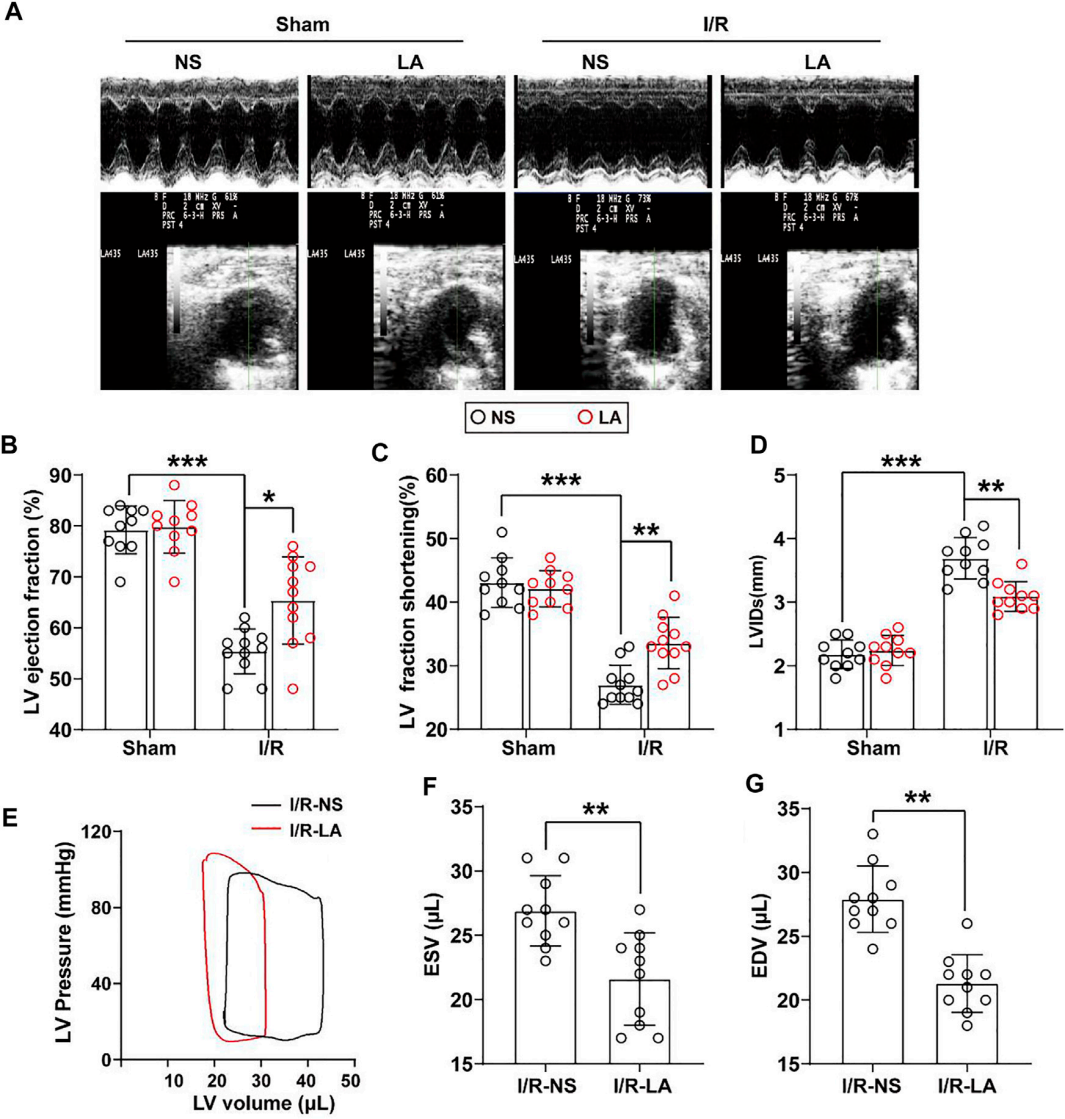
LA improves MI/R-induced cardiac dysfunction *in vivo.* LA and NS pretreated mice were subjected to MI/R operation or sham **(A)**. Representative M-mode and B-mode echocardiography of left ventricular chamber **(B–D)**. Measurement of left ventricle ejection fraction (LVEF), left ventricle fraction shortening (LVFS) and left ventricular end systolic diameter (LVIDs), *n* = 10–11 per group **(E)**. Representative PV loops of LA and NS pretreated mice following MI/R, and **(F,G)**. analysis of end systolic volume (ESV) and end diastolic volume (EDV) (n = 10). Data are presented as the mean ± SEM, with each point representing a mouse. * indicates *p* < 0.05, ** indicates *p* < 0.01, *** indicates *p* < 0.001.

To determine the effects of LA on cardiac function, echocardiography and hemodynamic assessment were performed in mice. It was found that LA attenuated MI/R-induced left ventricle systolic dysfunction (Figure 2A; Table 1). However, LA pretreatment partly restored LVEF, LVFS and LVIDs after MI/R surgery (Figures 2B–D). Moreover, LA administration significantly attenuated MI/R-mediated disturbance in hemodynamic parameters; the left ventricular pressure-volume (P-V) loop, end-systolic volume (ESV) and end-diastolic volume (EDV) remained relatively unchanged (Figures 2E–G). However, LA administration had a limited effect on hemodynamic parameters in sham mice (Supplementary Figure S1). Collectively, these data indicated that LA ameliorated MI/R-mediated cardiac dysfunction *in vivo*.

**TABLE 1:** Physiological, Echocardiographic and Hemodynamic parameters after MI/R.

	Sham-NS (n = 10) Mean ± SEM	Sham-LA (n = 10) Mean ± SEM	MI/R-NS (n = 14) Mean ± SEM	MI/R-LA (n = 15) Mean ± SEM
Physiological parameter				
HW (mg)	126.52 ± 7.24	128.36 ± 5.03	126.28 ± 4.96	129.63 ± 6.71
BW (g)	25.12 ± 1.63	24.85 ± 1.26	24.86 ± 3.38	25.03 ± 3.02
TL (mm)	18.24 ± 0.26	18.59 ± 0.15	18.46 ± 0.44	17.95 ± 0.52
HW/BW	4.95 ± 0.18	5.04 ± 0.23	4.96 ± 0.24	5.11 ± 0.42
HW/TL	6.98 ± 0.32	6.85 ± 0.39	6.93 ± 0.52	7.03 ± 0.47
Echocardiographic and Hemodynamic parameters				
HR, bpm	474 ± 17	467 ± 22	475 ± 24	464 ± 28
LVIDs, mm	2.35 ± 0.08	2.33 ± 0.11	3.71 ± 0.09*	3.09 ± 0.09#
IVSs, mm	0.69 ± 0.11	0.71 ± 0.09	0.81 ± 0.11*	0.75 ± 0.08#
EF (%)	80 ± 1	79 ± 1	54 ± 2*	64 ± 1#
FS (%)	43 ± 1	42 ± 2	26 ± 2*	32 ± 1#
CO (μL)	10,644 ± 3.56	10,574 ± 3.32	5,854 ± 3.39*	7,946 ± 3.51#
ESP (mmHg)	92 ± 1.9	91 ± 2.3	121 ± 2.5*	122 ± 2.7
dp/dt max (mmHg/s)	9,648 ± 116	9,736 ± 152	6,036 ± 106*	7,541 ± 126#
dp/dt min (mmHg/s)	**−**9,726 ± 129	**−**9,802 ± 96	−5,979 ± 103*	−7,265 ± 131#

Mean ± SEM., vs Sham-NS: *p < 0.05. vs MI/R-NS:^#^ *p* < 0.05.; Abbreviations: HW: heart weight/tibial length; BW: body weight; TL: tibial length; HR, heart rate; left ventricular end-systolic diameter; IVSs, interventricular septal thickness at end-systole; FS, fractional shortening; EF, ejection fraction; CO, cardiac output; ESP, end-systolic pressure.

### 3.3 LA Attenuates Oxidative Stress and Apoptosis in the Heart Following MI/R Surgery

**FIGURE 3:**
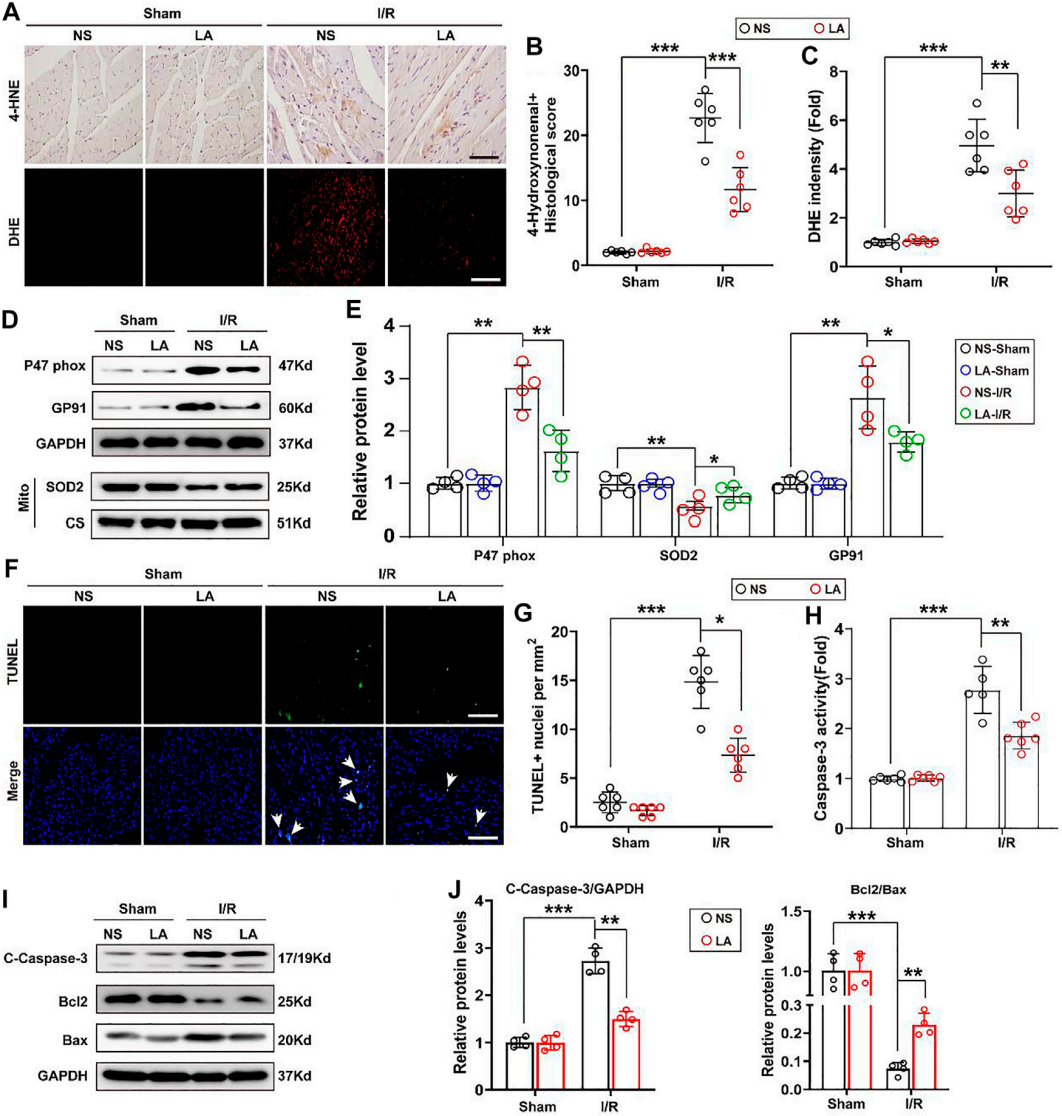
LA attenuates oxidative stress and apoptosis following MI/R operation in heart. LA and NS pretreated mice were subjected to MI/R operation or sham, and then hearts were harvested for histology and molecular analysis **(A–C)**. Representative immunohistochemistry of 4-hydroxynonenal and DHE staining in hearts, and intensity analysis (*n* = 6). Scar bar: 100 μm **(D,E).** Representative western blot and analysis of p47 phox, SOD2 and GP91 in hearts, normalized to GAPDH (*n* = 4) **(F,G)**. Representative Tunel and positive cells analysis in hearts (*n* = 6). Scar bar: 100 μm **(H).** Caspase-3 activity was tested in heart tissues (*n* = 6) **(I,J).** Representative western blot and analysis of cleaved-caspase-3 (C-caspase-3), Bax and Bcl2 in hearts, normalized to GAPDH (*n* = 4). Data are presented as the mean ± SEM, with each point representing a mouse. * indicates *p* < 0.05, ** indicates *p* < 0.01, *** indicates *p* < 0.001.

To evaluate whether LA regulates oxidative stress and apoptosis in the MI/R heart, immunohistochemistry staining of 4-hydroxynonenal (4-HNE) in the heart section was performed. The results showed a higher expression of 4-HNE after MI/R operation, which was significantly suppressed by LA pretreatment (Figures 3A,B). Similarly, DHE staining showed that LA pretreatment reduced MI/R-mediated ROS production (Figures 3A,C). Next, protein and transcriptional levels of oxidative stress markers were investigated. Immunoblot analysis revealed that p47 phox and GP91 were upregulated, whereas anti-oxidative marker SOD2 in mitochondria was decreased in response to MI/R in the heart. However, pretreatment with LA conferred protective effects against oxidative stress (Figures 3D,E). Likewise, RT-PCR analysis showed increased expression of oxidative factors such as GP91, NOX4 and p67 phox, while anti-oxidative markers such as Gpx, SOD2 and NQO1 were downregulated following MI/R surgery. However, LA treatment promoted the transcription of anti-oxidative genes and blocked the expression of oxidative genes in the heart (Supplementary Figure S2).

TUNEL staining was employed to determine the role of LA MI/R-induced apoptosis in heart tissues. TUNEL-positive cells were remarkably increased after MI/R surgery; however, LA treatment attenuated MI/R-induced apoptosis in the heart (Figures 3F,G). Subsequently, Caspase-3 activity was exanimated, and it was found that LA treatment reduced Caspase-3 activity following MI/R operation in the heart (Figure 3H). Similarly, immunoblotting analysis revealed that LA treatment alleviated MI/R-induced expression of cleaved Caspase-3 and Bax and contributed to Bcl2 production in the heart (Figures 3I,J). As expected, RT-PCR analysis revealed that LA treatment decreased mRNA levels of Bax and promoted transcription of Bcl2 in the heart (Supplementary Figure S3). These data suggested that LA treatment attenuated oxidative stress and apoptosis after MI/R surgery in the heart.

### 3.4 LA Counters Oxidative Stress and Apoptosis in Hypoxia Reoxygenation (HR) in H9C2 Cells

**FIGURE 4:**
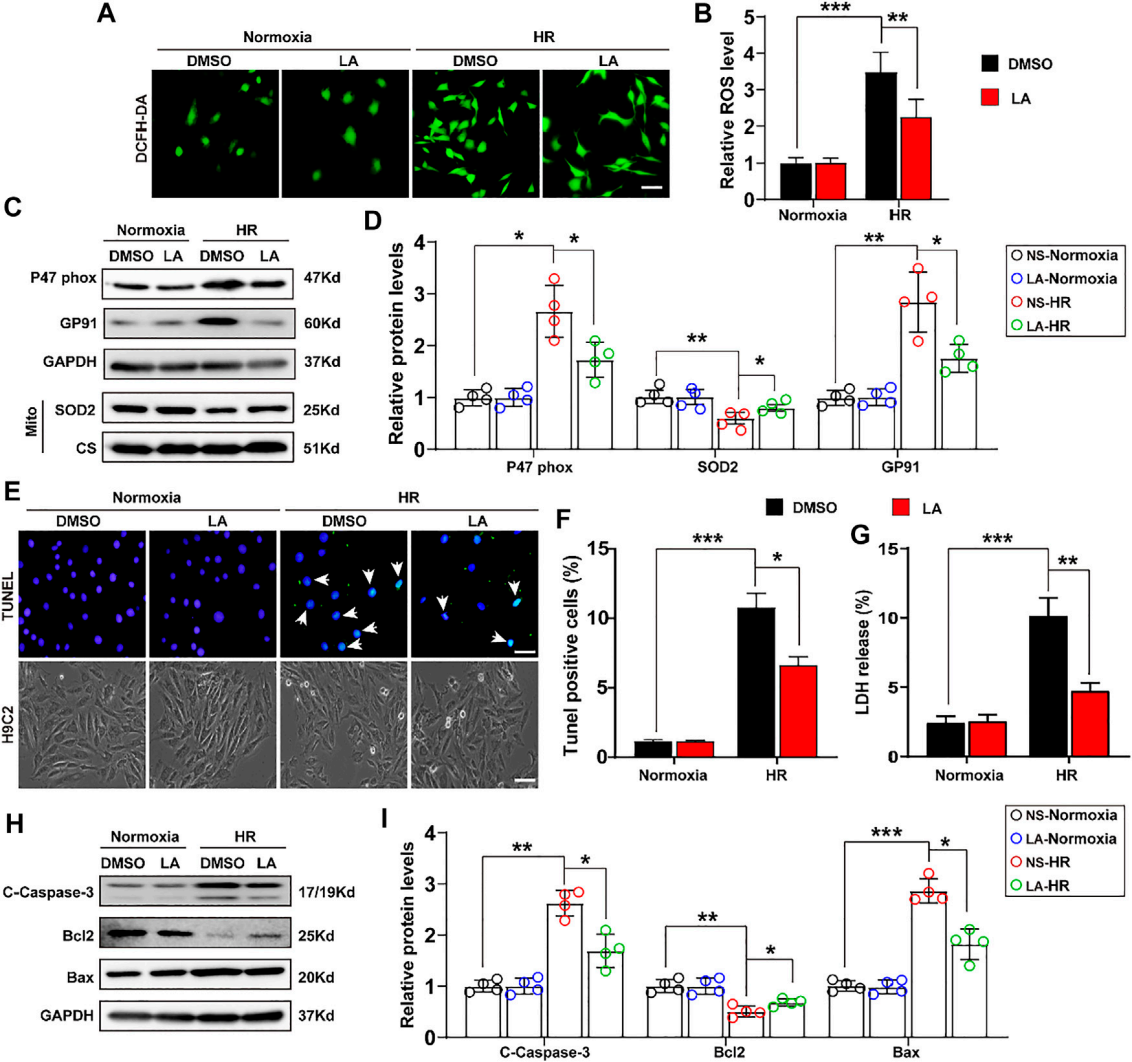
LA counters oxidative stress and apoptosis in hypoxia reoxygenation (HR) in H9C2 cells. LA and DMSO pretreated H9C2 cells were subjected to HR or Normoxia **(A,B)**. Representative ROS levels of H9C2 cells were measured by incubating with DCFH-DA probe, and then analysis fluorescence intensity, Scar bar: 50 μm **(C,D)**. Representative western blot and analysis of p47 phox, SOD2 and GP91 in H9C2 cells, normalized to GAPDH (n = 4) **(E,F)**. Representative Tunel and cell morphology, and positive cells analysis in H9C2 cells, Scar bar: 50 μm **(G).** LDH release was detected by LDH assay kits (H–I). Representative western blot and analysis of cleaved-caspase-3 (C-caspase-3), Bax and Bcl2 in H9C2 cells, normalized to GAPDH (*n* = 4). Data are presented as the mean ± SEM, with each point representing a mouse. * indicates *p* < 0.05, ** indicates *p* < 0.01, *** indicates *p* < 0.001.

To further explore the role of LA in H9C2 cells, *in vitro* experiments were performed with the HR model (Supplementary Figure S3). ROS levels were assessed using the 2′,7′-dihydro-dichlorofluorescein diacetate (DCFH-DA) probe in H9C2 cells. It was found that HR contributed to ROS production, whereas LA pretreatment decreased the expression of ROS in cardiomyocytes (Figures 4A,B). Similarly, Western blotting indicated that HR elevated the levels of p47 phox and GP91, and suppressed protein levels of SOD2 in H9C2 cells. Consistent with *in vivo* experiments, LA decreased protein levels of p47 phox and GP91 and promoted SOD2 production inside mitochondria in cardiomyocytes (Figures 4C,D). In addition, LA attenuated HR-induced upregulation of GP91 and p67 phox and triggered the transcription of SOD2 in H9C2 cells (Supplementary Figure S4).

Importantly, the extent of apoptosis in H9C2 cells following HR was also assessed. TUNEL staining showed that LA alleviated HR-induced apoptosis (Figures 4E,F). Subsequently, lactate dehydrogenase (LDH) release assay showed that HR resulted in the upregulation of LDH and LA protected against cell damage (Figure 4G). Consistent with *in vivo* experiments, LA decreased the expression of cleaved Caspase-3 and Bax and contributed to Bcl2 production in H9C2 cells following HR (Figures 4H,I). Moreover, LA prevented HR-induced upregulation of Bax and triggered the transcription of Bcl2 in H9C2 cells (Supplementary Figure S5).

### 3.5 LA Promotes the Expression of eNOS *in vivo and in vitro*

**FIGURE 5:**
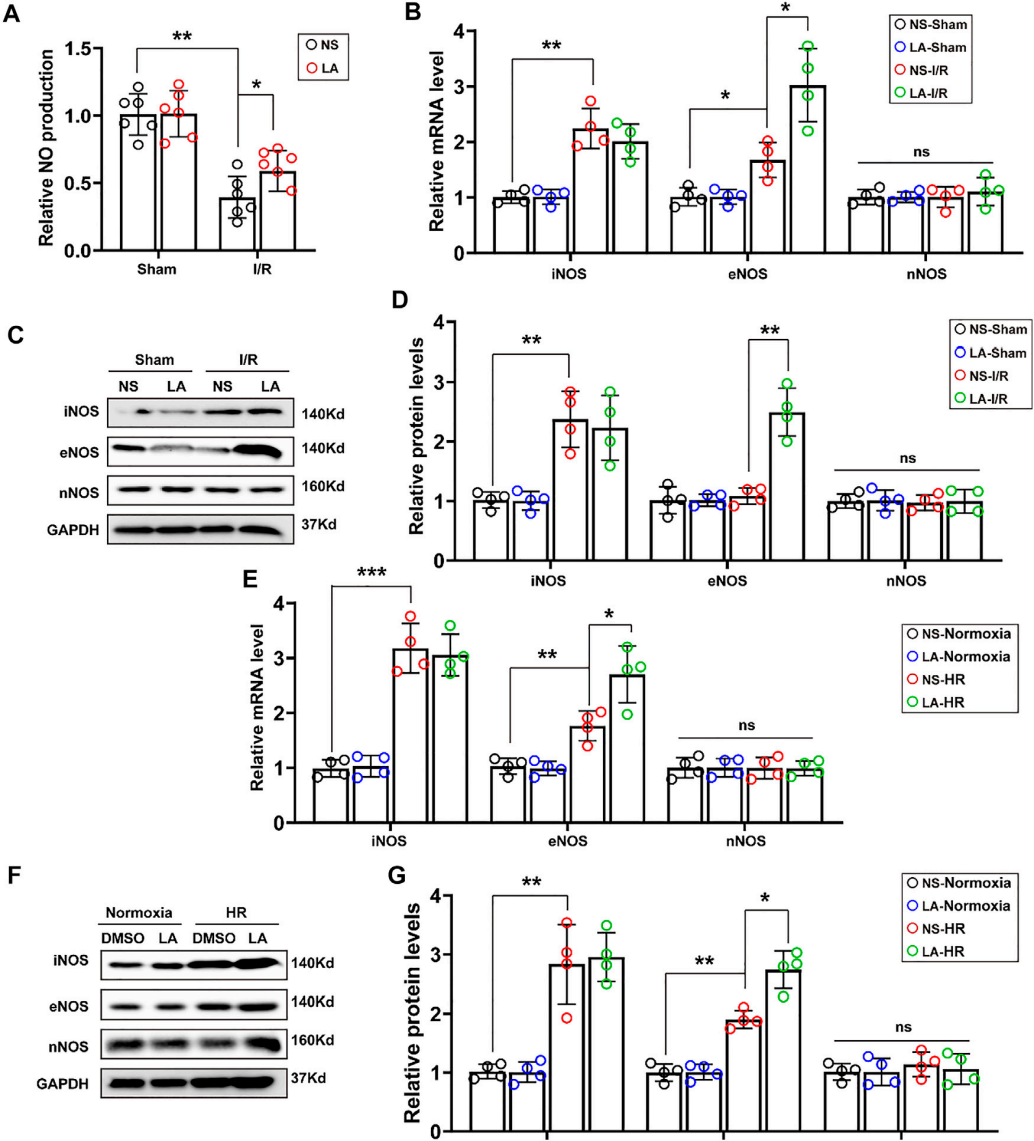
LA promotes expression of eNOS *in vivo and in vitro* **(A)**. NO production in the indicated groups 24 h post-MI/R operation (*n* = 6) **(B)**. The mRNA expression levels of iNOS, eNOS and nNOS in the indicated groups 24 h post-MI/R operation (*n* = 4). Normalized to GAPHD **(C,D)**. Representative western blot and analysis of iNOS, eNOS and nNOS in hearts following MI/R or sham, normalized to GAPDH (*n* = 4) **(E)**. The mRNA expression levels of iNOS, eNOS and nNOS in LA or DMSO pretreated H9C2 cells with or without HR **(F,G)**. Representative western blot and analysis of iNOS, eNOS and nNOS in H9C2 cells, normalized to GAPDH (*n* = 4). Data are presented as the mean ± SEM, with each point representing a mouse or a cell sample. * indicates *p* < 0.05, ** indicates *p* < 0.01, *** indicates *p* < 0.001, ns indicated no significance.

Furthermore, we investigated how LA alleviated oxidative stress and apoptosis. LA administration promoted nitric oxide (NO) production in heart tissues (Figure 5A), which was derived mainly from several isoforms of NO synthase (NOS), including neuronal NOS (nNOS), inducible NOS (iNOS) and eNOS isoforms (Crane et al., 2010). Therefore, the expression of iNOS, eNOS and nNOS in the heart was evaluated by RT-PCR. It was found that LA selectively activated eNOS (Figure 5B). Consistent Western blotting, LA increased the expression of eNOS but not iNOS and nNOS in the heart following MI/R surgery (Figures 5C,D). To assess the effects of LA on NOS in H9C2 cells, mRNA levels of the three isoforms of NOS in the HR model were measured by Western blotting and RT-PCR. Congruent with *in vivo* experiments, it was found that LA selectively increased transcription of eNOS in H9C2 cells following HR (Figure 5E), which was also observed in protein levels (Figures 5F,G). Overall, these data suggested that LA selectively promoted the expression of eNOS *in vivo* and *in vitro.*

### 3.6 LA Contributes to the Activation of the Nrf2/HO-1 Pathway and Nuclear Translocation of Nrf2 *in vivo and in vitro*

**FIGURE 6:**
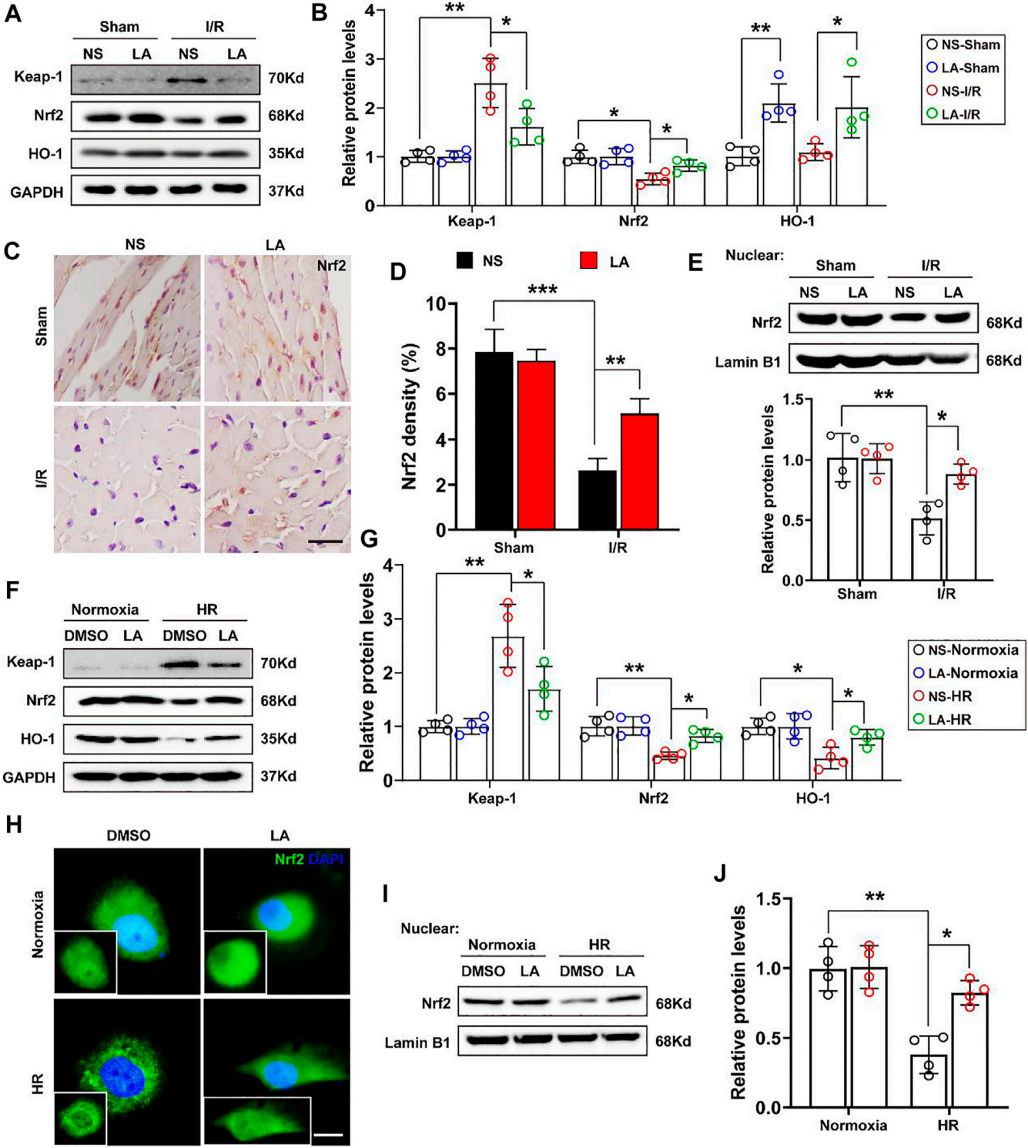
LA contributes to activation of Nrf2/HO-1 pathway and nuclear translocation of Nrf2 *in vivo and in vitro* **(A,B)**. Representative western blot and analysis of Keap-1, Nrf2 and HO-1 in hearts following MI/R or sham, normalized to GAPDH (*n* = 4) **(C,D)**. Representative immunohistochemistry and density analysis of Nrf2 in heart tissues (*n* > 25 from 3 mice per group). Scar bar: 100 μm **(E)**. Representative western blot and analysis of Nrf2 in nuclear from fresh heart tissues, normalized to Lamin B1 (*n* = 4) **(F,G)**. Representative western blot and analysis of Keap-1, Nrf2 and HO-1 in H9C2 cells, normalized to GAPDH (*n* = 4) **(H)**. Representative immunofluorescence of Nrf2 in LA or DMSO pretreated H9C2 cells with or without HR. Scar bar: 10 μm **(I,J)**. Representative western blot and analysis of Nrf2 in nuclear from H9C2 cells, normalized to Lamin B1 (*n* = 4). Data are presented as the mean ± SEM, with each point representing a mouse or a cell sample. * indicates *p* < 0.05, ** indicates *p* < 0.01, *** indicates *p* < 0.001.

To further elucidate how LA regulates oxidative stress, the role of Nrf2 signaling, a transcriptional coactivator that mediates anti-oxidative gene expression in the heart, was investigated (Dai et al., 2020). Immunoblot analysis revealed that MI/R largely contributed to the protein downregulation of Nrf2 and HO-1 in the heart; however, LA treatment restored the expression of the Nf2/HO-1 pathway (Figures 6A,B). The transcription activity of Nrf2 is reported to depend largely on the nuclear translocation of Nrf2 (Silva-Islas and Maldonado, 2018), Hence, the nuclear translocation of Nrf2 after MI/R and LA treatment was explored. IHC analysis revealed that MI/R reduced the levels of Nrf2 in the nuclear, and LA pretreatment partly restored the nuclear translocation of Nrf2 in the heart (Figures 6C,D). Subsequently, nuclear protein from fresh heart tissue was isolated and Western blotting showed that LA triggered nuclear levels of Nrf2 in response to MI/R (Figure 6E). Furthermore, the effects of LA on the Nrf2/HO-1 pathway *in vitro* were evaluated. Congruous with *in vivo* experiments, Western blotting showed that LA promoted the activation of the Nrf2/HO-1 pathway in H9C2 cells under HR (Figures 6F,G). Similarly, immunofluorescence of Nrf2 in H9C2 cells also showed that LA increased nuclear levels of Nrf2 in response to HR (Figure 6H). Additionally, Western blotting showed that LA promoted nuclear translocation of Nrf2 in H9C2 cells under HR (Figures 6I,J). In summary, these data suggested that LA contributed to the activation of Nrf2/HO-1 signaling and nuclear translocation of Nrf2 *in vivo* and *in vitro*.

### 3.7 LA-Mediated Activation of Nrf2/HO-1 Pathway Depends on the Phosphorylation of AMPK*α*

**FIGURE 7:**
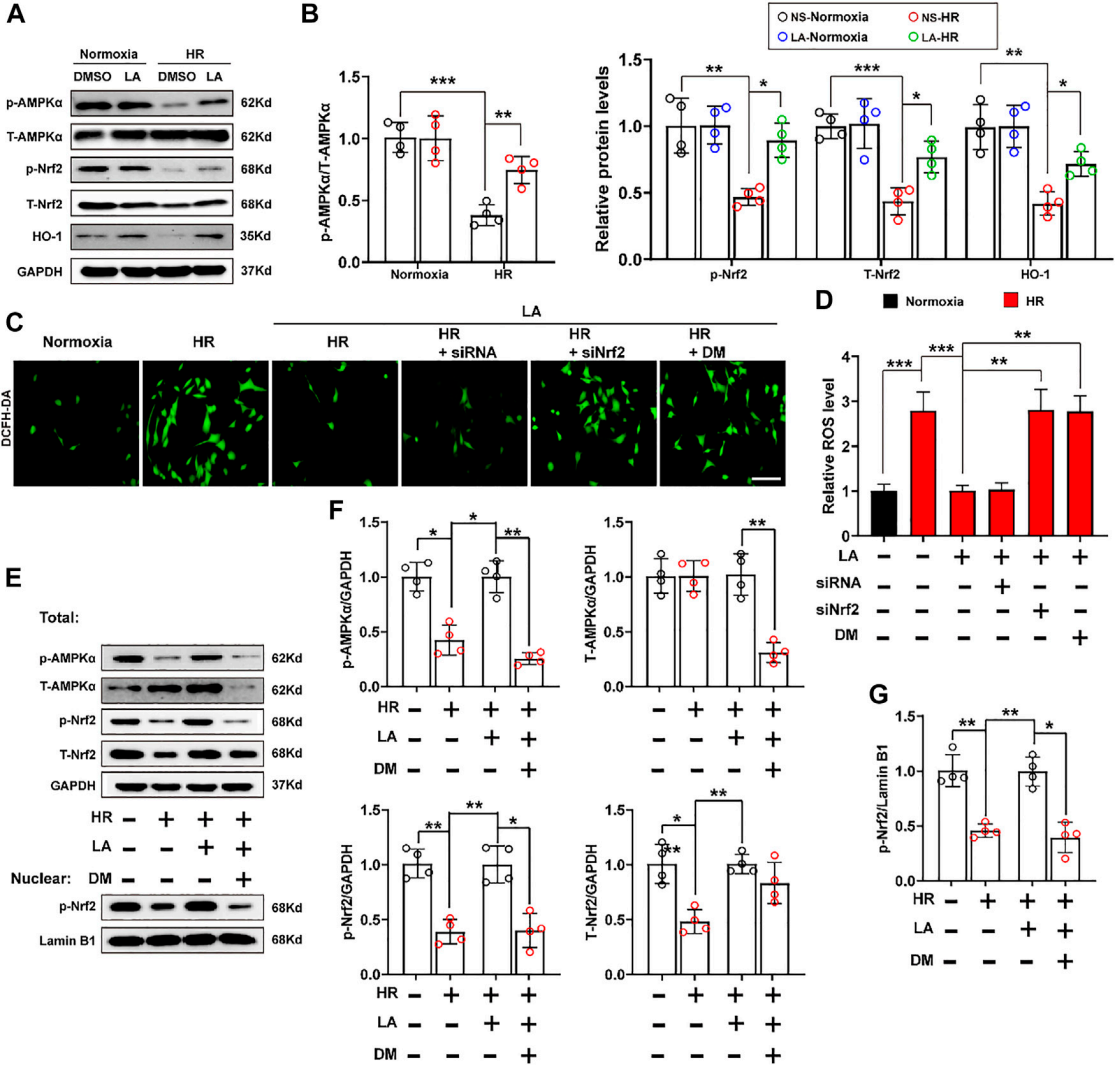
LA-mediated activation of Nrf2/HO-1 pathway depends on phosphorylation of AMPKα. Nrf2 siRNA and dorsomorphin, an inhibitor of AMPKα, LA and DMSO pretreated H9C2 cells were subjected to HR or Normoxia **(A,B)**. Representative western blot and analysis of p- AMPKα, AMPKα, *p*-Nrf2, Nrf2 and HO-1 in H9C2 cells, normalized to GAPDH (*n* = 4) **(C,D)**. Representative ROS levels of H9C2 cells were measured by incubating with DCFH-DA probe, and then analysis fluorescence intensity, Scar bar: 50 μm **(E,G)**. **(E)** and **(F)**. Representative western blot and analysis of p- AMPK*α*, AMPK*α*, *p*-Nrf2, Nrf2 and HO-1 in H9C2 cells, normalized to GAPDH (*n* = 4). **(E)** and **(G)**. Representative western blot and analysis of *p*-Nrf2 in nuclear from H9C2 cells, normalized to Lamin B1 (*n* = 4). Data are presented as the mean ± SEM, with each point representing a mouse or a cell sample. * indicates *p* < 0.05, ** indicates *p* < 0.01, *** indicates *p* < 0.001.

Previous studies reported that AMPK triggered phosphorylation of Nrf2 and promoted the transactivation of antioxidative genes (Matzinger et al., 2020). Herein, the effect of LA on the activation of AMPKα and phosphorylation of Nrf2 was further investigated. The results of Western blot showed that LA accelerated the activation and phosphorylation of AMPKα, which further resulted in phosphorylation of Nrf2 and activation of the Nrf2/HO-1 pathway *in vitro* (Figures 7A,B). To verify the role of Nrf2 and AMPKα in H9C2 cells, Nrf2 siRNA and DM, an inhibitor of AMPKα, were used to explore the function of AMPKα/Nrf2 in LA-mediated effects. We firstly tested ROS production in H9C2 cells using the DCFH-DA probe and found that LA-mediated blockage of ROS production was abolished by silencing Nrf2 or inhibiting AMPKα in H9C2 cells under HR (Figures 7C,D and Supplementary Figure S7A–D). Similarly, Western blotting revealed that inhibition of AMPKα decreased phosphorylated-modification and nuclear translocation of Nrf2 in H9C2 cells under HR (Figures 7E–G) and blockage of AMPKα countered LA-mediated upregulation of eNOS in response to HR (Supplementary Figure S7E,F). Overall, these data indicated that LA-mediated activation of Nrf2/HO-1 signaling and nuclear translocation of Nrf2 depended on the phosphorylation of AMPKα.

## 4 Discussion

Morbidity and mortality from AMI remain high, and reperfusion strategies are the current standard therapy for AMI. However, MI/R injury leads to increased oxidative stress and apoptosis, which further contribute to cellular injury, exacerbating the final infarct size (Toldo et al., 2018). Myocardial infarct size is a key factor of prognosis in patients with AMI. Therefore, cardioprotective strategies aim to reduce the infarct size (Heusch, 2020). In addition, LA has been considered as a potential antioxidant and anti-apoptotic drug (Kang et al., 2021). Herein, we found that LA pretreatment alleviated oxidative stress and apoptosis and improved cardiac function in the MI/R injury mouse model. LA may yield novel interventional strategies attenuating reperfusion injury in AMI. Lithospermic acid (LA) is a catechol derivative extracted from *Salvia miltiorrhiza*, which is a traditional Chinese herb widely used to treat multiple disorders (Shih et al., 2019). Liu et al. showed that LA is an oxidase inhibitor that strongly exerts anti-inflammatory and hypouricemic effects (Liu et al., 2008). Izabela et al. demonstrated that LA exhibited cytotoxicity against MCF-7 cell lines and suppressed breast cancer growth and metastasis (Berdowska et al., 2013). Furthermore, previous clinical trials showed that LA injection improved coronary heart diseases angina pectoris in a clinical trial (Zhang et al., 2006). The current study found that LA prevented cardiac dysfunction in MI/R injury, and may provide a potential therapeutic strategy for AMI treatment.

Recently, oxidative stress has been implicated in heart failure in different sources of stress and characterized by overproduction of ROS relative to anti-oxidant defenses (van der Pol et al., 2019; Aimo et al., 2020). Moreover, increased production of ROS caused cellular dysfunction such as lipid peroxidation, and increased DNA damage, resulting in cell death (Seddon et al., 2007). H In the present study, LA significantly blocked oxidative stress in the MI/R mouse model. Besides, as an important free radical, NO is synthesized by several NOS, and three isoforms of NOS are produced in the heart (Tang et al., 2014). Interestingly, both iNOS and eNOS were upregulated, and eNOS was unchanged in the MI/R heart. However, LA pretreatment increased eNOS but not iNOS or nNOS levels. Our previous study suggested that eNOS/Nrf2 pathway regulates pressure overload-induced cardiac remodeling (Liu et al., 2019). In addition, previous studies reported that the transcription activity of Nrf2 depends largely on the nuclear translocation of Nrf2 (Silva-Islas and Maldonado, 2018). Therefore, the effects of Nrf2 signaling on LA-mediated cardioprotection in MI/R injury were investigated. As anticipated, LA promoted the Nrf2/HO-1 pathway and contributed to the nuclear translocation of Nrf2 *in vivo* and *in vitro*.

As a member of the serine/threonine (Ser/Thr) kinase group, AMPK is widely distributed in various organs (Jiang et al., 2018). AMPK is closely associated with cellular metabolism and energy status, which is characterized by increased AMP/ATP and ADP/ATP ratios (Lin and Hardie, 2018). In addition, as a key controller of cellular homeostasis, AMPK plays a critical role in cardiovascular disease, diabetes and cancer (Qi and Young, 2015). A recent study suggested that AMPK-eNOS signaling regulated endothelial dysfunction and hypertension in the heart (Cheng et al., 2020). Moreover, AMPKα has been reported to be highly expressed in the heart (Fan et al., 2018). Therefore, the activation and phosphorylation of AMPKα were assessed. It was found that LA promoted phosphorylation of AMPKα and Nrf2 *in vivo* and *in vitro*, which corresponds with a recent study showing that AMPK triggered phosphorylation of Nrf2 and promoted transactivation of antioxidative genes (Matzinger et al., 2020). Furthermore, we demonstrated that LA-mediated activation of Nrf2/HO-1 pathway and nuclear translocation of Nrf2 depended on the phosphorylation of AMPK*α*. Although LA countered oxidative stress and apoptosis after MI/R by activating AMPKα/Nrf2 and eNOS pathway, evidence on direct interaction between AMPKα and Nrf2 warrants further exploration. Moreover, how AMPKα regulates eNOS should be further investigated.

In summary, this study demonstrates that LA improves cardiac function and attenuates myocardial injury in mice. It also suppresses oxidative stress and apoptosis following hypoxia-reoxygenation. Mechanistically, LA promotes the activation of the eNOS and Nrf2/HO-1 pathway by enhancing phosphorylation of AMPKα, providing a novel therapeutic strategy for reperfusion in AMI patients.

## Data Availability

The original contributions presented in the study are included in the article/Supplementary Material further inquiries can be directed to the corresponding author.

## Abbreviations

AMI, Acute myocardial infarction; AMPK*α*, AMP-activated protein kinase alpha; DHE, Dihydroethidium; DM, Dorsomorphin; eNOS, Endothelial nitric oxide synthase; 4-HNE, 4-Hydroxynonenal; HO-1, Heme oxygenase-1; HR, Hypoxia reoxygenation; LA, Lithospermic acid; MI/R, Myocardial ischemia and reperfusion; NS, Normal saline; Nrf2, Nuclear factor E2-related factor 2; ROS, Reactive oxygen species.
