# Supplementary material for: Activation of Nrf2 by Lithospermic Acid Ameliorates Myocardial Ischemia and Reperfusion Injury by Promoting Phosphorylation of AMP-Activated Protein Kinase α (AMPKα)
Source: Front Pharmacol. 2021 Nov 26;12:794982. doi: 10.3389/fphar.2021.794982 (PMC8661697; doi:10.3389/fphar.2021.794982)
Supplement: Supplementary file 2 [file DataSheet2.docx]

**Activation of Nrf2 by Lithospermic Acid Ameliorated Myocardial Ischemia and Reperfusion Injury by Promoting Phosphorylation of AMP-Activated Protein Kinase α (AMPKα)**

Running title: Lithospermic Acid Protects Against Myocardial I/R injury

Zhang et al.

**Address correspondence to:**

Qizhu Tang MD, PhD

Department of Cardiology,

Renmin Hospital of Wuhan University

Jiefang Road 238, Wuhan 430060, P.R. of China.

Tel.: +86 2788041911; Fax: +86 2788066234.

Email: [qztang@whu.edu.cn](mailto:qztang@whu.edu.cn)

*The data that support the findings of this study are available from the corresponding author upon reasonable request.*

**This PDF file includes:**

- **Visual Abstract and Highlights**
- **Supplementary Methods**
- **Supplementary Tables:**

**Supplementary Table 1.** Primary antibodies used in this study.

**Supplementary Table 2.** Primers used in qPCR.

- **Supplementary Figures and Figures Legends:**

**Supplementary Figure 1.** LA provided no effect on hemodynamic parameters in sham mice.

**Supplementary Figure 2.** LA pretreatment alleviates oxidative stress in mice following MI/R injury.

**Supplementary Figure 3.** LA pretreatment alleviates apoptosis in mice following MI/R injury.

**Supplementary Figure 4.** LA pretreatment blocks oxidative stress in H9C2 cells following hypoxia reoxygenation.

**Supplementary Figure 5.** LA pretreatment blocks apoptosis in H9C2 cells following hypoxia reoxygenation.

**Supplementary Figure 6.** Efficacy of Nrf2 siRNA in H9C2 cells.

**Supplementary Figure 7.** LA-mediated activation of Nrf2/HO-1 pathway depends on phosphorylation of AMPKα.

- **Supplementary References**
- **Visual Abstract and Highlights**


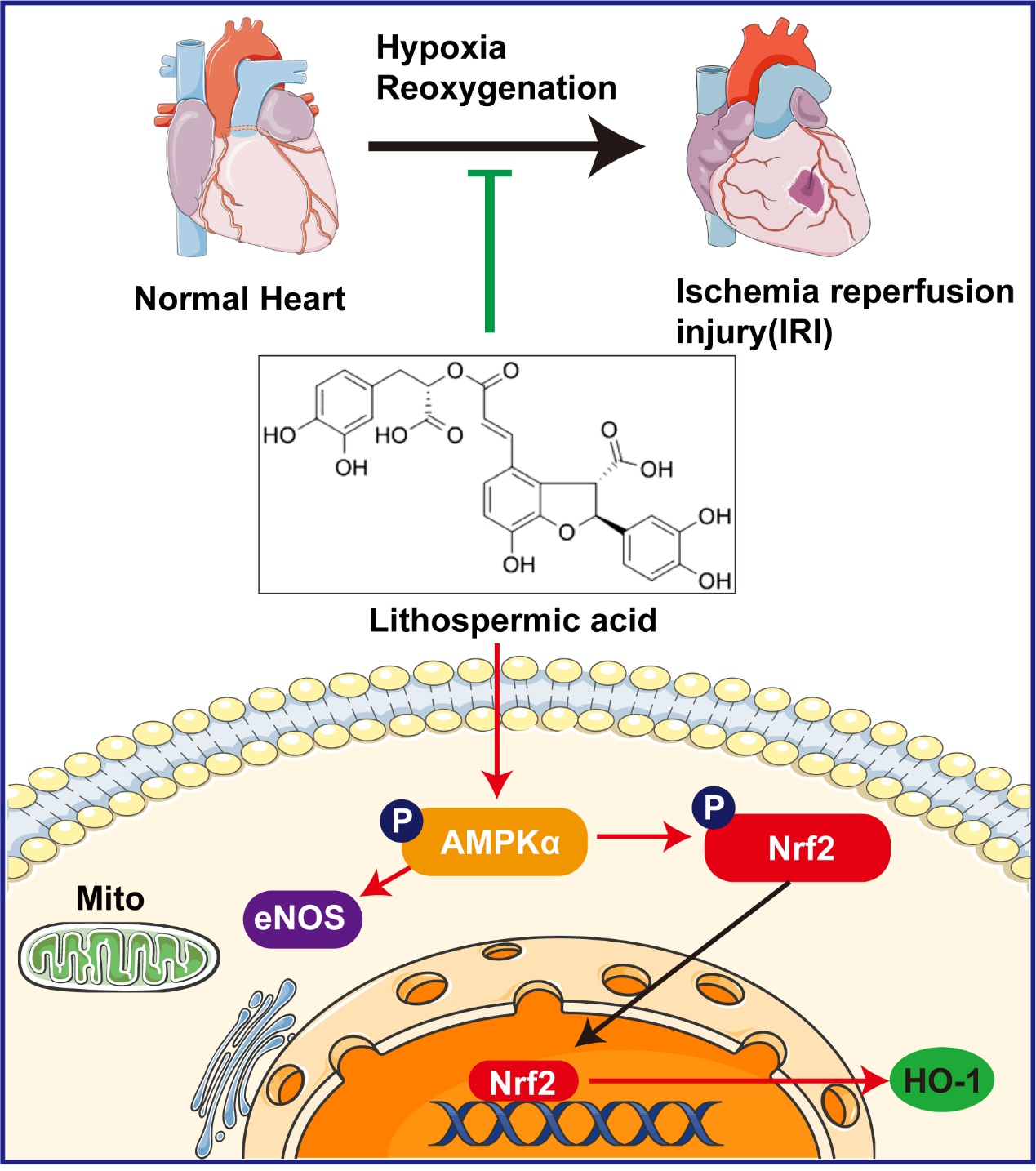


**Visual Abstract and Highlights**

- Lithospermic Acid improves cardiac function and attenuates myocardial injury during myocardial ischemia and reperfusion injury.
- Lithospermic Acid suppresses oxidative stress and apoptosis following hypoxia reoxygenation *in vivo and in vitro*.
- Lithospermic Acid promotes activation of eNOS and Nrf2/HO-1 pathway by enhancing phosphorylation of AMPKα.
- **Supplementary methods**

**Measure of area at risk and infarcted area**

2,3,5-triphenyltetrazolium chloride (TTC), purchased from Sigma (USA, T8877), was dissolved in 37℃ PBS. Evans-Blue (Sigma, E2129) was dissolved in normal saline to 5.0%. After echocardiography and hemodynamics detection, mice were subjected to 1mL 5.0% Evans-Blue by orbital intravenous injection. At this time, the Evans-blue dye covered the damaged myocardium through the reperfusion blood flow, and dyed the ischemia part in red and white in the infarcted part. Subsequently, hearts were harvested until the extremities and tail tips turn into blue, and heart tissues were quickly put in liquid nitrogen. Then hearts were cut into 5 equal parts along the direction perpendicular to the mandrel and put into preheated TTC solution for 10 min.

**Immunohistochemistry (IHC) staining**

For immunohistochemistry, the heart sections were heated using the pressure cooker method for antigen retrieval, incubated with anti-Nrf2 (12721, CST) or 4-hydroxynonenal (ab46545, Abcam) followed by incubation with goat anti-rabbit EnVisionTM+/ horseradish peroxidase (HRP) reagent, and stained using a DAB detection kit. The immunohistochemical positive area in the entire heart cross-section is used for statistics, and the heart section from sham mice was as normalized. And the microscope was Aperio VERSA (Leica). The intensity was measured by using a quantitative digital image analysis system (Image-Pro Plus, version 6.0).

**TUNEL staining**

The terminal deoxynucleotidyl transferase-mediated dUTP nick end-labelling (TUNEL) staining was performed according to the manufacturer’s instructions using a commercially available kit (Millipore, USA) to detect apoptosis. After that, the sections were mounted with DAPI, and were observed under the OLYMPUS DX51 fluorescence microscope (Tokyo, Japan). All the of images were quantified by Image-Pro Plus, version 6.0.

**Small interfering RNA transfection**

For adenovirus-mediated overexpression and small interfering RNA (siRNA)-mediated knockdown experiments. NRF2-siRNA or Scr-siRNA was transfected with Lipofectamine 6000 (lipo6000) at 40 nM concentration in culture medium according to the manufacturer’s protocol. The cell lysate was used to access the efficiency of overexpression or knockdown by western blot.

**Detection of ROS in cardiomyocytes**

Myocytes were cultured in 6-well plates and pretreated with LA and HR for the indicated times. ROS were then detected by dichlorofluorescein diacetate assay (DCFH-DA). The cells were incubated with DCFH-DA (10 μM) for 60 min at 37°C, and immunofluorescence was detected using a fluorescence microplate reader normalized to the vehicle-PBS group to control for unwanted sources of variation (excitation wavelength/emission wavelength: 485/525 nm), by light microscopy (Olympus Corporation, Tokyo, Japan).

**NO production**

NO production was determined as the amount of nitrate plus nitrite using the Griess reaction assay (Cayman Chemical, Ann Arbor, MI) according to the manufacturer’s instructions, as previously described ^1^.

**Mitochondrial proteins isolation**

Isolation of mitochondria was performed as the instruction of Mitochondrial Isolation Kit (Beyotime, C3606 and C3601). Briefly, mouse heart ventricles or cell lysates were collected, minced, and incubated with trypsin before homogenization with a glass/teflon Potter Elvehjem homogenizer once. Ventricle homogenates were centrifuged at 800 g at 4℃ for 10 min; the supernatant was collected and centrifuged at16,000 g at 4℃ for 10 min; the resulting pellet containing normal- and small-size mitochondria were washed once and centrifuged at 16, 000 g at 4℃ for 10 min. After re-suspension, mitochondrial protein concentrations were calorimetrically measured using protein assay dye reagent concentrate (Bio-Rad, 500-0006) and subsequent mitochondrial functional assays were performed.

**LDH release assay**

LDH assay kits (Nanjing Jiancheng Bioengineering Institute, Nanjing, China) were used in accordance with the manufacturer’s instructions to detect LDH activity. After incubated with LA in Normoxia or HR, the cells were centrifuged in tubes to obtain the supernatant. The ratio of cell number to extract was 500-1000: 1, ultrasound was used to lyse the cells, the absorbance was measured at 490 nm, as previously described ^2^.

**ELISA**

The antioxidants SOD, MDA, and GSH in myocardium were analyzed using the SOD assay kit (Beyotime, China; Cat. No. 19160), MDA assay kit (Beyotime, China; Cat. No. 19475) and GSH assay kit (Beyotime, China; Cat. No. 15367) as our previously described ^1^. The activity of caspase‐3 was detected using the Caspase‐3 Assay Kit (Sigma; Cat. No. CASP3C), the content of myocardial enzyme profile plasma concentrations of troponin T (TnT), creatine Kinase Isoenzyme-MB (CK-MB) were detected by TnT assay kit (Nanjing jiancheng, China; Cat. No. 1014), CK-MB assay kit (Nanjing jiancheng, China; Cat. No. 1036), according to the manufacturer's instructions.

**Data and statistical analysis**

All data in this study were presented as mean ± standard error of the mean (SEM) and were evaluated by linear mixed modeling (IBM SPSS Statistics, version 22). One-way analysis of variance (ANOVA) followed by Tukey post hoc test was used when comparing multiple groups, and differences between two groups were evaluated by unpaired Student’s t-test. A P-value<0.05 was considered statistically significant.

- **Supplementary Tables.**

**Supplementary Table 1.** Primary antibodies used in this study

**Table S1. Primary antibodies used in this study**

| **Antibody** | **Customer** | **Product number** | **Dilution** | **Application** |
| --- | --- | --- | --- | --- |
| 4-hydroxynonenal | Abcam | Ab46545 | 1:200 | IHC |
| P47 phox | CST | 4312 | 1:1000 | WB |
| SOD2 | Abcam | Ab68155 | 1:1000 | WB |
| GP91 | Abcam | Ab129068 | 1:1000 | WB |
| C-caspase-3 | CST | 9661 | 1:1000 | WB |
| Bcl2 | Abcam | Ab196495 | 1:1000 | IF |
| Bax | CST | 2772 | 1:1000 | WB |
| iNOS | Abcam | Ab15323 | 1:1000 | IF |
| GAPDH | CST | 2118 | 1:1000 | WB |
| nNOS | CST | 4321 | 1:1000 | WB |
| eNOS | CST | 32027S | 1:1000 | WB |
| p-Nrf2 | Invitrogen | PA5-67520 | 1:1000 | WB |
| p-AMPKα | CST | 2535 | 1:1000 | WB |
| Lamin B1 | Abcam | Ab16048 | 1:1000 | WB |
| Citrate synthetase | Abcam | Ab129095 | 1:1000 | WB |
| Nrf2 | CST | 12721 | 1:1000  1:200  1:200 | WB  IHC  IF |
| AMPKα | CST | 2603P | 1:1000 | WB |
| Keap-1 | CST | 4678 | 1:1000 | WB |
| HO-1 | Abcam | Ab13243 | 1:1000 | WB |

**Supplementary Table 2.** Primers and probes used in quantitative RT-PCR

**Table S2. Primers Used in qPCR**

| **Species** | **Gene** | **Forward Primer (5’-3’)** | **Reverse Primer (5’-3’)** |
| --- | --- | --- | --- |
| Mouse | *inos* | TGCCAGGGTCACAACTTTACA | CTCTCCACTGCCCCAGTTTT |
| Mouse | *enos* | ATCTTGAAGGTTCCTCCGGC | ACTGGGTTACAGAGAGGTGTC |
| Mouse | *nnos* | ACAACCCTGCCATCACTAGC | GCAGAGGGCAGTATCGACTC |
| Mouse | *Gpx* | GAGAATGGCAAGAATGAAGAG | GAAGGTAAAGAGCGGGTGA |
| Mouse | *Gp91* | TTCCAGTGCGTGTTGCTCGACA | TGGCGGTGTGCAGTGCTATCAT |
| Mouse | *P67* | GCCGGAGACGCCAGAAGAGCTA | GGGGCTGCGACTGAGGGTGAA |
| Mouse | *Nox4* | ATGTTGGGCCTAGGATTGTGTT | GGCTACATGCACACCTGAGA |
| Mouse | *Sod2* | CCGTCCGTGTCGCCGTCCTC | GCCGCGTGGTGCTTGCTGTG |
| Mouse | *Nqo1* | CCAATCAGCGTTCGGTATTA | GTCTTCTCTGAATGGGCCAG |
| Mouse | *Bcl2* | CTTTGAGTTCGGTGGGGTCA | CCAGAATCCACTCACACCCC |
| Mouse | *Bax* | CTGGATCCAAGACCAGGGTG | CCTTTCCCCTTCCCCCATTC |
| Mouse | *Gapdh* | GCATCTTCTTGTGCAGTGCC | TACGGCCAAATCCGTTCACA |
| Rat | *inos* | GCAGGGCCACCTCTATGTTT | TGGTCACCCAAAGTGCTTCA |
| Rat | *enos* | GGTTGACCAAGGCAAACCAC | CCTAATACCACAGCCGGAGG |
| Rat | *nnos* | CTGCAGCCTCGCTACTACTC | AGCCGTGTGTGTCCGAATTT |
| Rat | *SOD2* | AGCCTCCCTGACCTGCCTTA | CGCCTCGTGGTACTTCTCCTC |
| Rat | *Gp91* | TGAATCTCAGGCCAATCACTTT | AATGGTCTTGAACTCGTTATCCC |
| Rat | *Gpx* | AGTGCGAGGTGAATGGTGAG | CGCCCATCTGAGGGGATTTT |
| Rat | *Bax* | CACGTCTGCGGGGAGTCA | TAGGAAAGGAGGCCATCCCA |
| Rat | *Bcl2* | GGTGAACTGGGGGAGGATTG | AGAGCGATGTTGTCCACCAG |
| Rat | *Gapdh* | GCATCTTCTTGTGCAGTGCC | GATGGTGATGGGTTTCCCGT |

- **Supplementary Figures and Figure Legends:**

**Suppl Figure 1.**

**
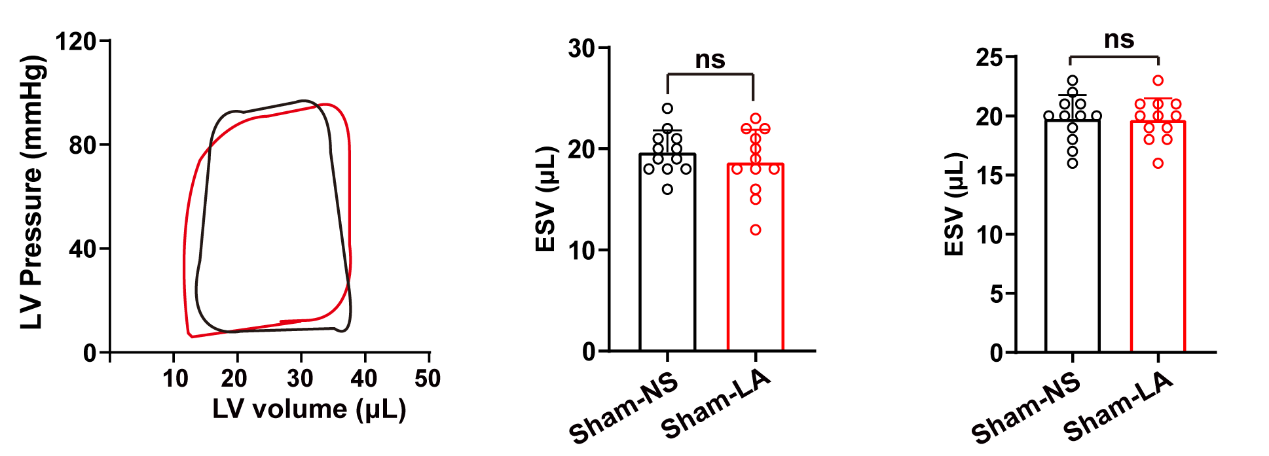
**

**Suppl Figure 1. LA provided no effect on hemodynamic parameters in sham mice.**

Representative PV loops of LA and NS pretreated mice following Sham, and analysis of end systolic volume (ESV) and end diastolic volume (EDV) (n =12 per group). Data are presented as the mean±SEM, with each point representing a mouse. ns indicates no significance.

**Suppl Figure 2.**


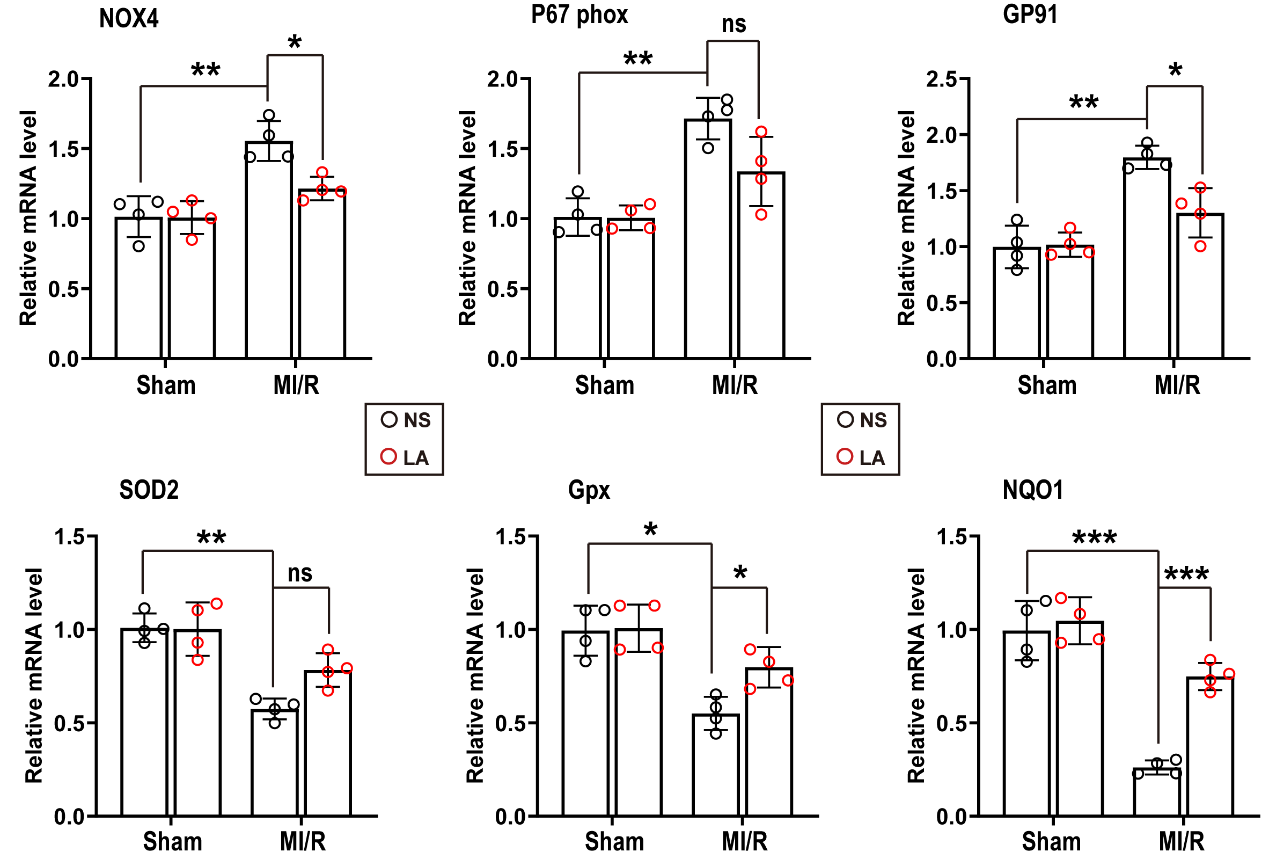


**Suppl Figure 2.** **LA pretreatment alleviates oxidative stress in mice following MI/R injury.** RNA was harvested from MI/R or sham mice with or without LA treatment, and then mRNA levels of Nox4, p67, GP91, SOD2, Gpx and NQO1 were detected by RT-PCR. Normalized to GAPDH. (n=4 per group). Data are presented as the mean±SEM, with each point representing a heart sample. * indicates p<0.05, **** indicates p<0.01, ***** indicates p<0.001, ns indicates no significance.

**Suppl Figure 3.**


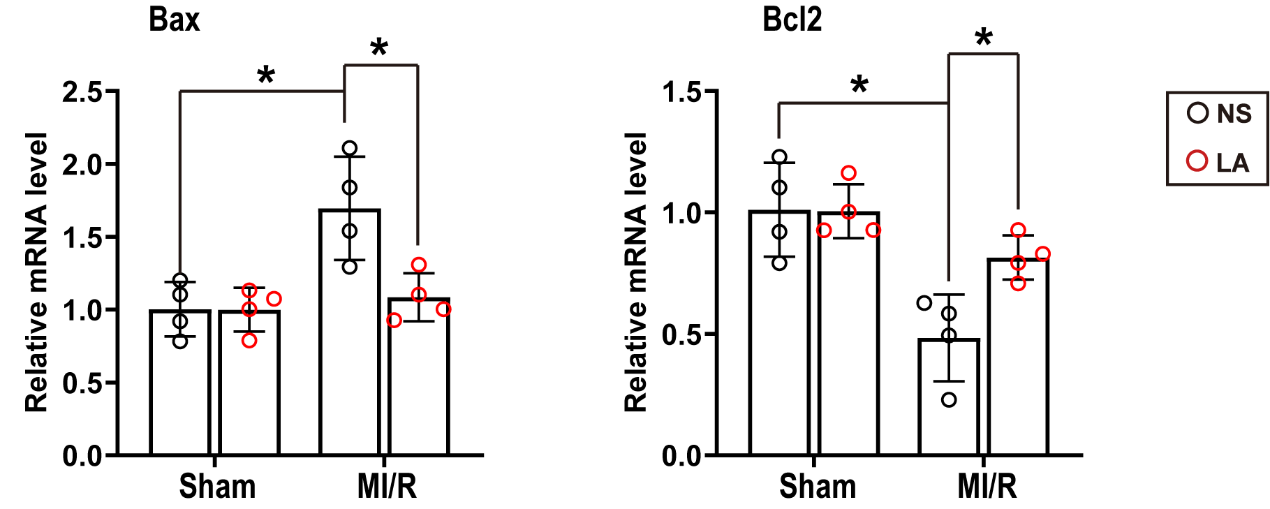


**Suppl Figure 3. LA pretreatment alleviates apoptosis in mice following MI/R injury.** RNA was harvested from MI/R or sham mice with or without LA treatment, and then mRNA levels of Bax and Bcl2 were detected by RT-PCR. Normalized to GAPDH. (n=4 per group). Data are presented as the mean±SEM, with each point representing a heart sample. * indicates p<0.05.

**Suppl Figure 4.**


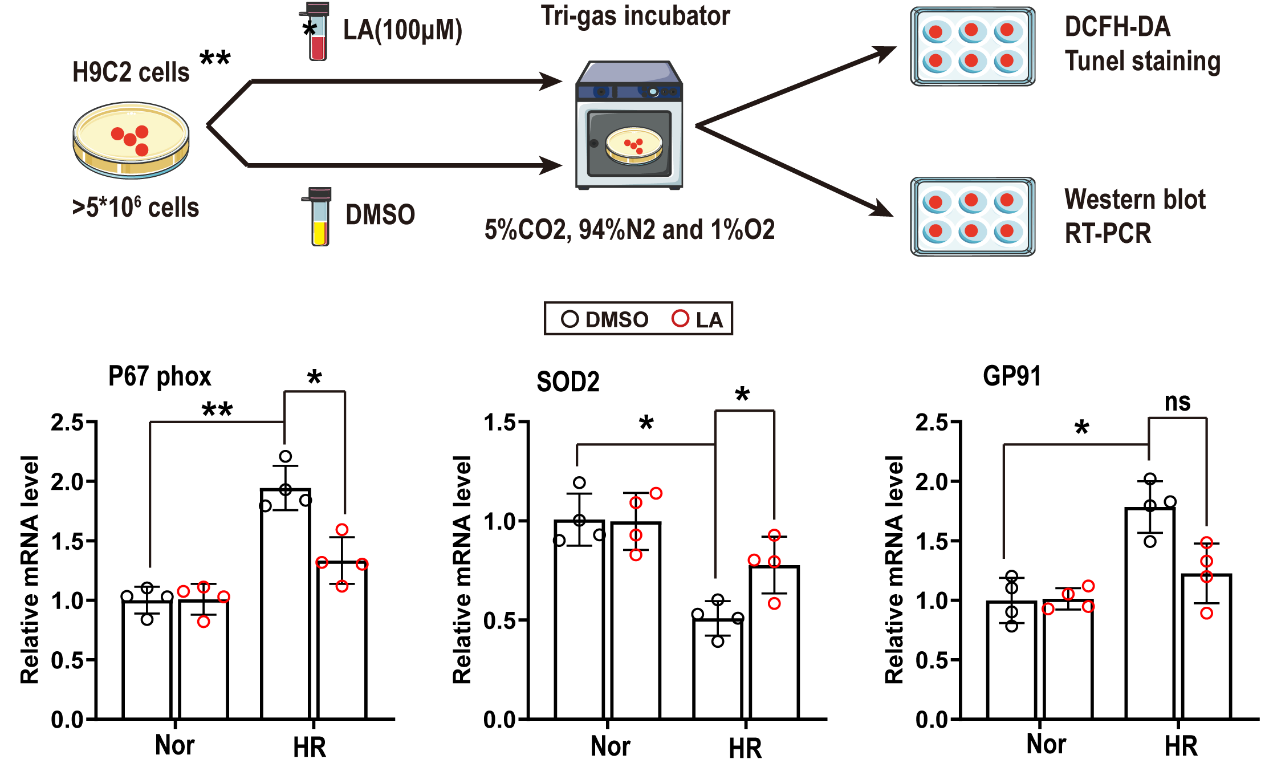


**Suppl Figure 4. LA pretreatment blocks oxidative stress in H9C2 cells following hypoxia reoxygenation.** Protocol *in vitro*. RNA was harvested from Normoxia or HR H9C2 cells with or without LA treatment, and then mRNA levels of p67, GP91 and SOD2 were detected by RT-PCR. Normalized to GAPDH. (n=4 per group). Data are presented as the mean±SEM, with each point representing a heart sample. * indicates p<0.05, **** indicates p<0.01, ns indicates no significance.

**Suppl Figure 5.**


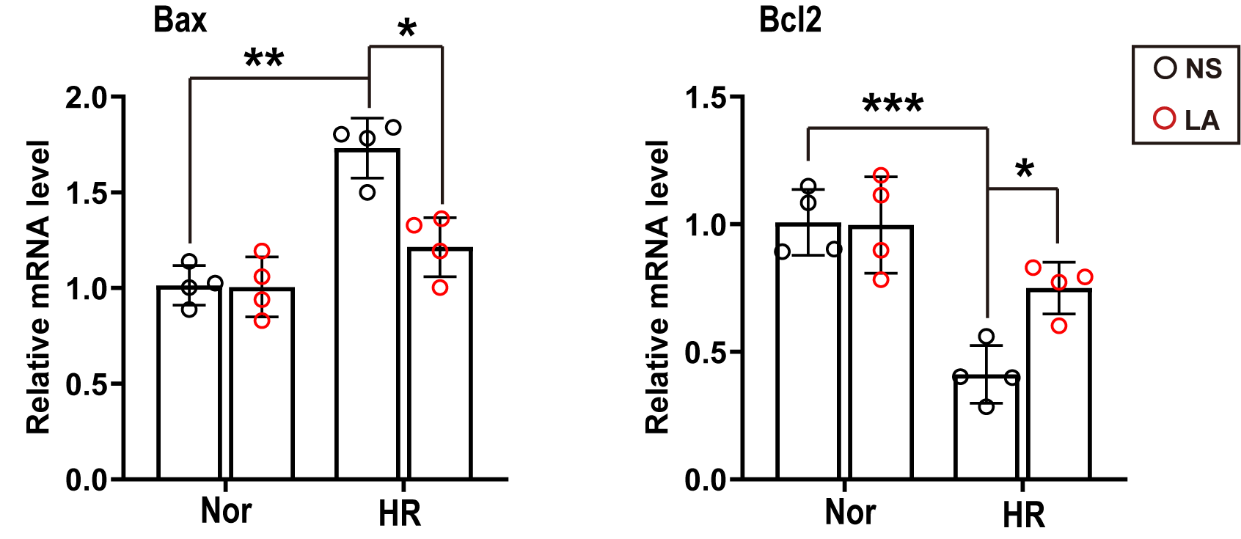


**Suppl Figure 5. LA pretreatment blocks apoptosis in H9C2 cells following hypoxia reoxygenation.** RNA was harvested from Normoxia or HR H9C2 cells with or without LA treatment, and then mRNA levels of Bax and Bcl2 were detected by RT-PCR. Normalized to GAPDH. (n=4 per group). Data are presented as the mean±SEM, with each point representing a cell sample. * indicates p<0.05, **** indicates p<0.01, ***** indicates p<0.001.

**Suppl Figure 6.**

**
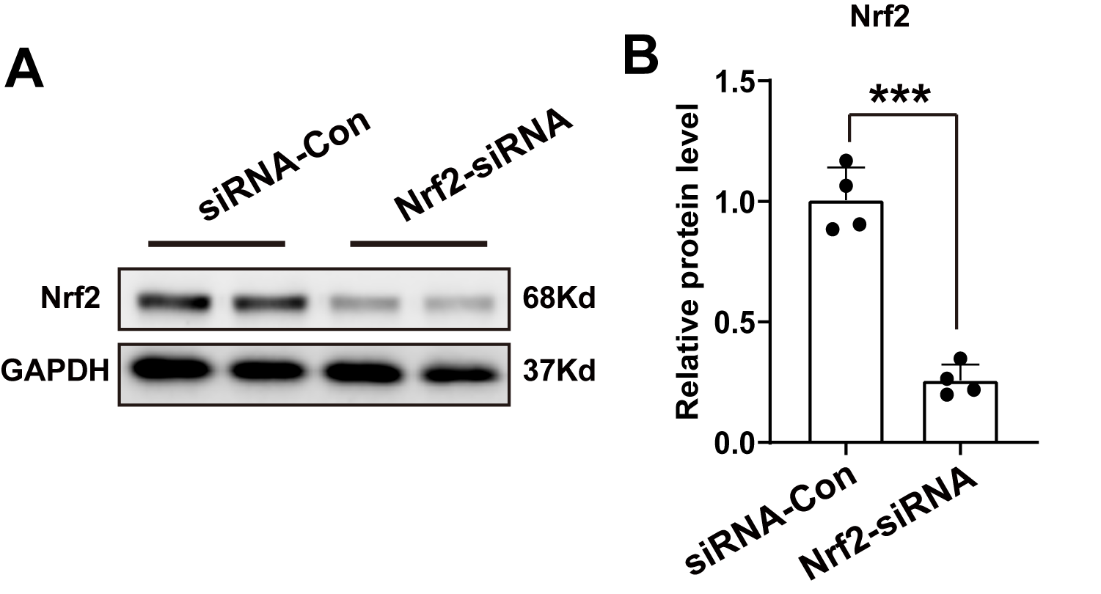
**

**Suppl figure 6. Efficacy of Nrf2 siRNA in H9C2 cells.**

**A-B.** The knockdown efficacy of Nrf2 siRNA in H9C2 cells was accessed by western blot and representative analysis (n=4 per group). Data are presented as the mean±SEM, with each point representing a cell sample. *** indicates p<0.001.

**Suppl Figure 7.**

**
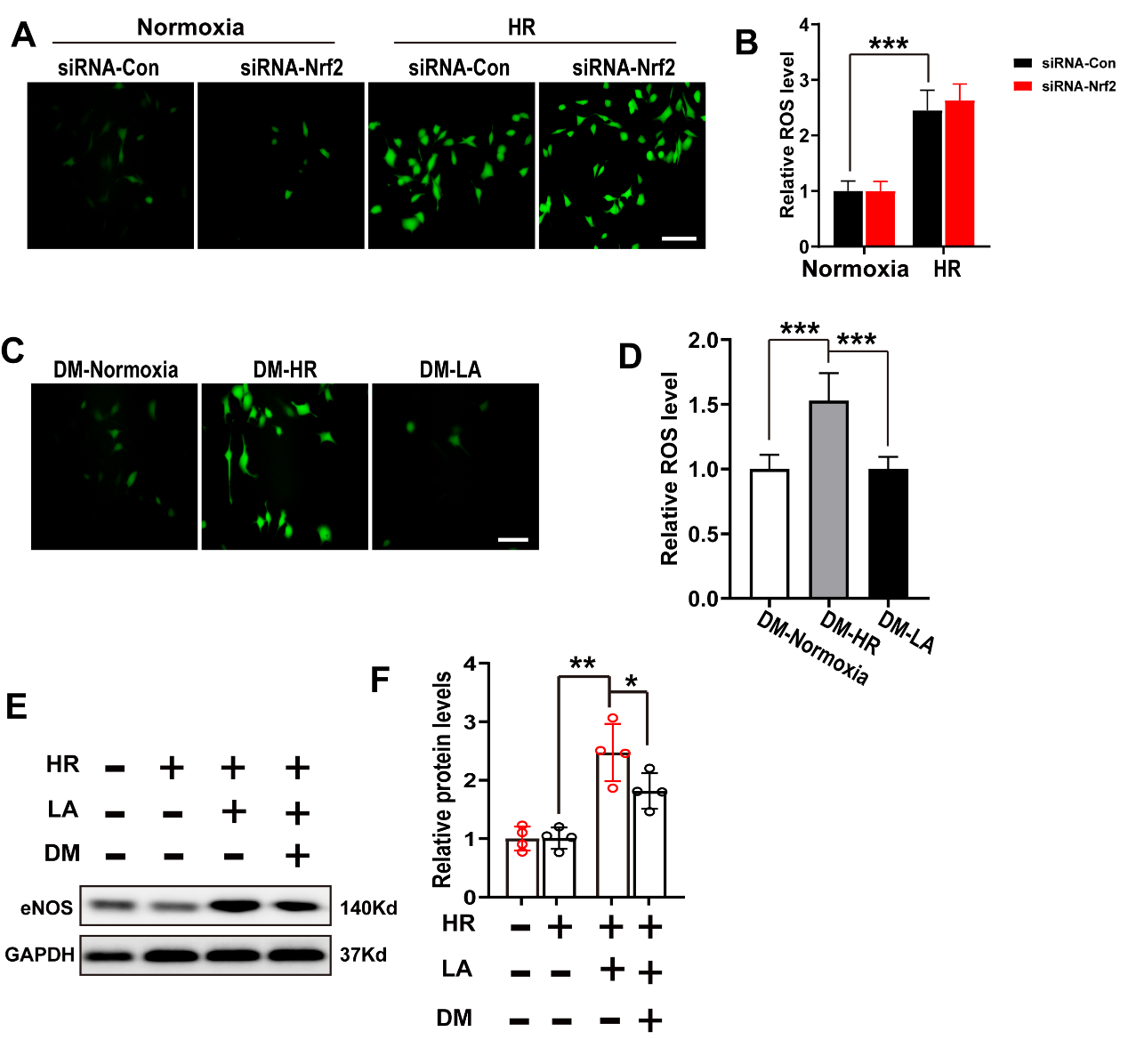
**

**Suppl Figure 7. LA-mediated activation of Nrf2/HO-1 pathway depends on phosphorylation of AMPKα. A-D.** Representative ROS levels of H9C2 cells were measured by incubating with DCFH-DA probe, and then analysis fluorescence intensity, Scar bar: 50 μm. **E-F**. Representative western blot and analysis of eNOS in H9C2 cells, normalized to GAPDH (n=4 per group). Data are presented as the mean±SEM, with each point representing a mouse or a cell sample. * indicates p<0.05, ** indicates p<0.01, ***** indicates p<0.001.

- **Supplementary Reference**

1. Wu, Q.Q., Y. Xiao, M.X. Duan, Y. Yuan, X.H. Jiang, Z. Yang, et al., *Aucubin protects against pressure overload-induced cardiac remodelling via the beta3 -adrenoceptor-neuronal NOS cascades.* Br J Pharmacol, 2018; 175:1548-1566.

2. Duan, M., Y. Yuan, C. Liu, Z. Cai, Q. Xie, T. Hu, et al., *Indigo Fruits Ingredient, Aucubin, Protects against LPS-Induced Cardiac Dysfunction in Mice.* J Pharmacol Exp Ther, 2019; 371:348-359.
